# The DNA Repair Gene *APE1* T1349G Polymorphism and Risk of Gastric Cancer in a Chinese Population

**DOI:** 10.1371/journal.pone.0028971

**Published:** 2011-12-19

**Authors:** Dongying Gu, Meilin Wang, Shizhi Wang, Zhengdong Zhang, Jinfei Chen

**Affiliations:** 1 Department of Oncology, The Affiliated Nanjing First Hospital, Nanjing Medical University, Nanjing, China; 2 Department of Molecular & Genetic Toxicology, School of Public Health, Nanjing Medical University, Nanjing, China; IFOM, Fondazione Istituto FIRC di Oncologia Molecolare, Italy

## Abstract

**Background:**

Apurinic/apyrimidinic endonuclease 1 (*APE1*) has a central role in the repair of apurinic apyrimidic sites through both its endonuclease and its phosphodiesterase activities. A common *APE1* polymorphism, T1349G (rs3136820), was previously shown to be associated with the risk of cancers.

**Objective:**

We hypothesized that the *APE1* T1349G polymorphism is also associated with risk of gastric cancer.

**Methods:**

In a hospital-based case-control study of 338 case patients with newly diagnosed gastric cancer and 362 cancer-free controls frequency-matched by age and sex, we genotyped the T1349G polymorphism and assessed its associations with risk of gastric cancer.

**Results:**

Compared with the *APE1* TT genotype, individuals with the variant TG/GG genotypes had a significantly increased risk of gastric cancer (odds ratio = 1.69, 95% confidence interval = 1.19–2.40), which was more pronounced among subgroups of aged ≤60 years, male, ever smokers, and ever drinkers. Further analyses revealed that the variant genotypes were associated with an increased risk for diffuse-type, low depth of tumor infiltration (T1 and T2), and lymph node metastasis gastric cancer.

**Conclusions:**

The *APE1* T1349G polymorphism may be a marker for the development of gastric cancer in the Chinese population. Larger studies are required to validate these findings in diverse populations.

## Introduction

Gastric cancer is the fourth most common cancer and the second leading cause of cancer-related death worldwide, approximately 700,000 people die of this malignancy each year, and around 1.1 million new cases are expected in 2010 [1]. In China, it was predicted to rank as the third most common cancer in 2005, with 0.3 million deaths and 0.4 million new cases from gastric cancer [2]. Although it is well known that environmental factors, dietary habits, and *Helicobacter pylori* infection are associated with the risk of gastric cancer, host genetic factors may be one of the most critical agents in gastric carcinogenesis. Presentation of gastric cancer in young patients is frequently associated with familial clustering [3].

DNA damage induced by exogenous carcinogens or by endogenous metabolic processes can be converted into gene mutations leading to genomic instability and malignant transformation [4]. Genetic variations in DNA repair genes can modulate DNA repair capacity and, consequently, alter cancer risk. About 150 human DNA repair genes have been identified to data [5]. They are cooperating in distinct pathways that are specialized in the repair of the different types of DNA damage [6], [7]. Among these pathways, the base excision repair (BER) pathway, which possibly handles the largest number of cytotoxic and mutagenic base lesions, has been associated with risk of cancers [8].

The human apurinic/apyrimidinic endonuclease (APE), *APE1* (also known as *APE*, *APEX*, and *REF-1*), is involved in the BER pathway [9]. The *APE1* gene is located on chromosome 14q11.2-q12, and consists of five exons spanning 2.21 kb. By hydrolyzing 3′-blocking fragments from oxidized DNA, *APE1* produces normal 3′-hydroxyl nucleotide termini that are necessary for DNA repair synthesis and ligation at single- or double-strand breaks [10], [11]. A total of 18 polymorphisms in *APE1* have been reported [12], but the most extensively studied polymorphism is the T to G transversion (T1349G, also known as Asp148Glu, rs3136820).

Recently, we have carried out a meta-analysis on all eligible case-control studies to estimate the *APE1* T1349G polymorphism and risk of cancers, including lung cancer, bladder cancer, colorectal cancer, breast cancer, pancreatic cancer, head and neck cancer, leukaemia, esophageal cancer, biliary tract cancer, thyroid cancer, and prostate cancer [13]. We found that the *APE1* T1349G variant genotypes were associated with a moderately increased risk of all cancer types (OR = 1.08, 95%CI = 1.00–1.18 in a dominant model), suggesting that this polymorphism is a low-penetrance risk factor for cancer development [13]. More recently, some studies reported the *APE1* T1349G polymorphism was associated with gastric cancer risk and prognosis [14], [15], [16], [17]. However, these epidemiologic results remain conflicting rather than conclusive.

In the present study, to investigate the effect of the *APE1* T1349G polymorphism on the risk of gastric cancer, we genotyped the polymorphism and evaluated the association between the *APE1* T1349G polymorphism and the risk of gastric cancer in our ongoing, hospital-based, case-control study in a Chinese population.

## Materials and Methods

### Ethics Statement

The ethics committee of Nanjing Medical University has approved the research protocol. During epidemiological interviews, written informed consent was given to all subjects. Furthermore, trained research staff administered a standard questionnaire to obtain information on demographic characteristics.

### Study subjects

The detailed methods of recruiting study subjects for this study have been described previously [18]. Briefly, 338 newly diagnosed, histopathologically confirmed gastric cancer patients and 362 cancer-free control subjects were recruited from the cities of Yangzhong and Yixin, two regions of high incidence and mortality rate of gastric cancer in Jiangsu Province of China between March 2006 and July 2008. The exclusion criteria included previous cancer, metastasized cancer from other or unknown origins, and previous radiotherapy or chemotherapy. Controls were frequency-matched to the cases by age (±5 years) and sex. The cancer-free control subjects were recruited from those who were seeking health care, living in the same residential areas. Before recruitment, informed consent was obtained from each of the eligible subjects. For each individual, demographic information as well as data on smoking status and alcohol use was obtained through face-to-face interviews. Individuals who smoked once a day for more than 1 year were defined as ever smokers. Ever smokers who had quit smoking for more than 1 year were defined as former smokers, and the other smokers as current smokers. Individuals who consumed one or more alcoholic drinks per week for at least 1 year were considered ever drinkers, and the rest were defined as never drinkers. After interview, about 5 ml venous blood sample was collected from each participant.

### Genotyping

Genomic DNA was extracted from peripheral blood lymphocytes by proteinase K digestion, followed by phenol-chloroform extraction and ethanol precipitation [19]. The *APE1* T1349G polymorphism was determined using the polymerase chain reaction-restriction fragment length polymorphism (PCR-RFLP) method, which has been described previously [20], [21]. We designed the primer of 5′-TAATTCTGTTTCATTTCTATAGGCTA-3′ (forward) and 5′-TGCATTAGGTACATATGCTGTT-3′ (reverse) to amplify the target fragment of *APE1*. Samples were subjected to denaturation at 95°C for 5 min, followed by 30 cycles of heating at 95°C for 30 s, annealing at 55°C for 40 s, then extension at 72°C for 45 s, and a final incubation at 72°C for 10 min. The PCR products of 108 bp were digested by the *FspB*I restriction enzyme, to identify genotypes of the T1349G polymorphism. After the enzyme digestion, the variant G allele produced 2 fragments of 82-bp and 20-bp, and the wild-type T allele resulted in a single 108-bp fragment.

The genotype analyses were performed by two persons independently in a blind fashion. About 10% of the samples were randomly selected for confirmation, and the results were fully concordant.

### Statistical analysis

Differences between cases and controls in selected demographic variables, smoking status, alcohol use, and each allele and genotype of the polymorphisms of *APE1* gene were evaluated using the Chi-square test. Chi-square test was also used to assess the difference between the *APE1* polymorphism and clinicopathologic characteristics. The associations between *APE1* genotypes and the risk of gastric cancer were estimated by computing the odds ratios (ORs) and their 95% confidence intervals (CIs) from unconditional univariate and multivariate logistic regression analyses. These analyses were repeated by stratifying for sex (male, female), smoking status (never, ever) and drinking status (no, yes). For stratification by age, the age cutoff at 60 years was used in this study as it was the median age of the recruited patients and controls. Furthermore, the genotypic-specific risks were also estimated separately for cardia/noncardia tumor site, intestinal/diffuse histotype, T1/T2/T3/T4 depth of tumor infiltration and negative/positive lymph node metastasis. Hardy-Weinberg equilibrium of the controls' genotype distributions was tested by a goodness-of-fit chi-square test. Two-sided tests of statistical significance were conducted by using the SAS software (version 8.2; SAS Institute, Inc., Cary, NC).

## Results

Distribution of selected characteristics between the gastric cancer cases and control subjects are summarized in Table 1. According to the genotyping results and questionnaire data, we included 338 gastric cancer cases and 362 cancer-free controls who had all these data available in this analysis. The mean age at diagnosis for the gastric cancer cases was 61.7 years (standard deviation (SD)±12.3) and mean age at ascertainment for the controls was 62.4 years ((SD)±10.5). There were no statistically significant differences between cases and controls in the distribution of age and sex. However, higher incidences of smoking and drinking are associated with gastric cancer (*P*<0.05). Furthermore, patients with cancer of the gastric cardia and non-cardia were 143 (42.3%) and 195 (57.5%), respectively. The histological types were 192 (56.8%) for intestinal and 146 (43.2%) for diffuse gastric cancer. For the depth of tumor infiltration, T1, T2, T3, and T4 were 104 (30.8%), 64 (18.9%), 126 (37.3%), and 44 (13.0%), respectively. And positive lymph nodes were identified in 174 (51.5%) cases.

**Table 1:** Distribution of selected variables between the gastric cancer cases and control subjects.

Variables	Cases (*n* = 338)		Controls (*n* = 362)		*P*
	*n*	%	*n*	%	
Age (years) (mean ± SD)	61.7±12.3		62.4±10.5		0.469^a^
Sex					
Male	222	65.7	239	66.0	0.924^b^
Female	116	34.3	123	34.0	
Smoking status					
Never	182	53.9	231	63.8	0.007^b^
Ever	156	46.1	131	36.2	
Drinking status					
No	212	62.7	258	71.3	0.016^b^
Yes	126	37.3	104	28.7	
Tumor site					
Cardia	143	42.3			
Non-cardia	195	57.5			
Histological type					
Intestinal	192	56.8			
Diffuse	146	43.2			
Depth of tumor infiltration					
T1	104	30.8			
T2	64	18.9			
T3	126	37.3			
T4	44	13.0			
Lymph node metastasis					
Negative	164	48.5			
Positive	174	51.5			

a Two-sided t-test for difference between the cases and controls.

b Two-sided χ^2^ test for distribution between the cases and controls.

The genotype and allele frequencies of the *APE1* T1349G polymorphism among the cases and controls and the associations with risk of gastric cancer are shown in Table 2. The frequencies of the TT, TG, and GG genotypes were 20.4%, 54.7%, and 24.9% in the cancer group, versus 30.4%, 50.5%, and 19.1% in normal healthy individuals, respectively. The frequency of G allele was 52.2% among cases, which was significantly greater than that in controls 44.3% (*P* = 0.003). A subsequent analysis revealed a significant association of heterozygous (TG) and homozygous (GG) variant genotype of *APE1* T1349G with the risk of gastric cancer (OR = 1.61, 95% CI = 1.12–2.32 for TG versus TT, and OR = 1.92, 95% CI = 1.23–2.99 for GG versus TT, respectively). In addition, we found that individuals with the variant TG/GG genotypes had a significantly higher risk of gastric cancer than those with the TT genotype (OR = 1.69, 95% CI = 1.19–2.40). Further stratified analyses showed that the increased risk was more evident in subgroups of aged≤60 years (OR = 2.00, 95% CI = 1.11–3.61), male (OR = 1.65, 95% CI = 1.07–2.55), ever smokers (OR = 2.00, 95% CI = 1.14–3.05), and ever drinkers (OR = 2.18, 95% CI = 1.18–4.03) (Table 3). However, because of the limited study sample size, all the results from stratified analyses were preliminary.

**Table 2:** Genotype and allele frequencies of the *APE1* T1349G polymorphism among the cases and controls and the associations with risk of gastric cancer.

*APE1* T1349G	Cases (*n* = 338)		Controls (*n* = 362)		OR (95% CI)^a^	*P*
	*n*	%	*n*	%		
TT	69	20.4	110	30.4	1.00 (ref.)	
TG	185	54.7	183	50.5	1.61 (1.12–2.32)	0.011
GG	84	24.9	69	19.1	1.92 (1.23–2.99)	0.004
TG/GG	269	75.6	252	69.6	1.69 (1.19–2.40)	0.003

a Adjusted for age, sex, smoking, and alcohol status in logistic regression model.

**Table 3:** Stratified analyses for the *APE1* T1349G polymorphism in gastric cancer cases and control subjects.

Variables	TT genotype		TG/GG genotypes		TG/GG vs. TT
	Cases	Controls	Cases	Controls	OR (95% CI)^a^
	N (%)	N (%)	N (%)	N (%)	
Age (years)					
≤60	26 (18.3)	35 (31.5)	116 (81.7)	76 (68.5)	2.00 (1.11–3.61)
>60	43 (21.9)	75 (29.9)	153 (78.1)	176 (70.1)	1.49 (0.96–2.31)
Sex					
Male	45 (20.3)	71 (29.7)	177 (79.7)	168 (70.3)	1.65 (1.07–2.55)
Female	24 (20.7)	39 (31.7)	92 (79.3)	84 (68.3)	1.72 (0.95–3.12)
Smoking status					
Never	41 (22.5)	71 (30.7)	141 (77.5)	160 (69.3)	1.50 (0.96–2.36)
Ever	28 (18.0)	39 (29.8)	128 (82.0)	92 (70.2)	2.00 (1.14–3.05)
Drinking status					
No	45 (21.2)	73 (28.3)	167 (78.8)	185 (71.7)	1.47 (0.96–2.25)
Yes	24 (19.1)	37 (35.6)	102 (80.9)	67 (64.4)	2.18 (1.18–4.03)

a Adjusted for age, sex, smoking, and alcohol status in logistic regression model.

Association between the *APE1* T1349G polymorphism and clinicopathologic characteristics of gastric cancer risk stratified on tumor site, histological type, depth of tumor infiltration and lymph node metastasis are shown in Table 4. Statistical analysis revealed a significant association of TG/GG genotypes with the risk of cardia tumor and non-cardia tumor (OR = 1.61, 95% CI = 1.01–2.56, and OR = 1.79, 95% CI = 1.17–2.73, respectively). Statistically, a trend toward a higher risk of diffuse-type gastric cancer was detected for TG/GG genotypes (OR = 2.09, 95% CI = 1.29–3.42), but there was no significant difference between intestinal-type gastric cancers and normal controls (OR = 1.46, 95% CI = 0.97–2.20). Further stratification analysis of depth of tumor infiltration showed that the increased risk was more pronounced between T1 (OR = 2.73, 95% CI = 1.50–4.99) and T2 (OR = 2.17, 95% CI = 1.08–4.33), but there were no significant differences between distributions in subgroups of T3 and T4 (OR = 1.28, 95% CI = 0.80–2.04, and OR = 1.26, 95% CI = 0.61–2.60, respectively). In addition, there were a significant increased risk in node-positive gastric cancer (OR = 1.80, 95% CI = 1.16–2.80), while not in node-negative gastric cancer (OR = 1.62, 95% CI = 0.99–2.51) with the TG/GG genotypes compared with the TT genotype.

**Table 4:** Association between the *APE1* T1349G polymorphism and clinicopathologic characteristics of gastric cancer risk.

*APE1* T1349G	Genotype		TG/GG vs. TT	
	TT	TG/GG	*P*	OR (95% CI)^a^
Controls (*n* = 362)	110	252		1.00 (ref.)
Cases (*n* = 338)	69	269		
Tumor site				
Cardia	43	151	0.046	1.61 (1.01–2.56)
Non-cardia	26	118	0.007	1.79 (1.17–2.73)
Histological type				
Intestinal	44	148	0.072	1.46 (0.97–2.20)
Diffuse	25	121	0.003	2.09 (1.29–3.42)
Depth of tumor infiltration				
T1	15	89	0.001	2.73 (1.50–4.99)
T2	11	53	0.029	2.17 (1.08–4.33)
T3	32	94	0.303	1.28 (0.80–2.04)
T4	11	33	0.537	1.26 (0.61–2.60)
Lymph node metastasis				
Negative	35	129	0.051	1.62 (0.99–2.51)
Positive	34	140	0.009	1.80 (1.16–2.80)

a Adjusted for age, sex, smoking, and alcohol status in logistic regression model.

## Discussion

In this hospital-based case-control study, we investigated the association of *APE1* T1349G polymorphism and risk of gastric cancer in Chinese populations. We found that the T1349G polymorphism contributed to the risk of gastric cancer, which was more pronounced among subgroups of aged≤60 years, male, ever smokers, and ever drinkers. Furthermore, the T1349G polymorphism was associated with an increased risk for diffuse-type, low depth of tumor infiltration (T1 and T2), and lymph node metastasis gastric cancer.

APE1 is an essential enzyme in the BER pathway, which is the primary mechanism for the repair of DNA damage caused by oxidation and alkylation [7]. The T1349G polymorphism is the most common polymorphism that result in single amino acid substitution has been identified in the general population [12]. It has not been determined if the G variant allele has an impact on endonuclease and DNA binding activities [22]. However, the GG genotype has been associated with significantly prolonged cell cycle G2 delays compared with the TT and TG genotypes, which suggests that this amino acid substitution may contribute to hypersensitivity to ionizing radiation [23]. Our study found that the G variant allele was associated with a significantly increased risk of gastric cancer, which is consistent with our previously published meta-analysis results [13]. In previous studies, Canbay et al. [15] found significant differences in the frequencies of G allele of *APE1* T1349G polymorphism between gastric cancer patients and control subjects in a Turkish population. Nevertheless, Palli et al. [16] did not observe significant association of this polymorphism with gastric cancer in their study. The reason for these different findings remains unclear, which might due to small sample sizes or ethnically diverse.

In the addition, we found that the T1349G polymorphism was associated with increased risk of gastric cancer among subgroups of younger subjects (age≤60 years) but not older subjects. Weak immune system and overwhelming accumulated exposure to environmental carcinogens in older individuals may account for the age difference we observed [24]. The older individuals are at a higher risk of gastric cancer, which is more likely due to the aging effect rather than direct genetic effects. Therefore, the variation in the *APE1* gene may be more influential in early-onset gastric cancer, although this result needs more confirmations. Tobacco smoke contains hundreds of chemicals, which was unequivocally established as the main causative factor for gastric cancer [25]. Our results indicated that the risk associated with the T1349G polymorphism was more evident in smokers. This may be because cigarette smoke generates reactive oxygen species production and induces DNA adducts [26]. Studies have reported that increase alcohol intake may increase risk of cancer, including gastric cancer [27]. Consistently, our results also revealed that subjects who have ever consumed alcohol with the T1349G polymorphism have an increased risk of gastric cancer. However, our small sample size might not have a sufficient power to detect the significant gene-environment interaction; thus, larger studies with more detailed environmental exposure data are needed to verify these findings.

Intestinal and diffuse types have different epidemiology and pathogenesis. The intestinal-type gastric cancer is international variation and predominates in high-risk geographic areas, especially in Japan, Korea and China, whereas the diffuse-type gastric cancer has uniform geographic distributions [28], [29]. Environmental factors have been proposed to play an important role in the etiology of intestinal-type gastric cancer, and the genetic susceptibility factors may contributed more to diffused-type gastric cancer development. Interestingly, we found that the effect of the T1349G polymorphism was associated with diffused-type gastric cancer but not intestinal-type gastric cancer. Furthermore, we found that the T1349G polymorphism was associated with an increased risk for low depth of tumor infiltration (T1 and T2), and lymph node metastasis gastric cancer, which may serve as a biomarker of gastric cancer metastasis. However, because the detectable effect size in our study was relatively small (n = 11 for TT genotype in the T1 and T2 subgroups), the clinical utility of our findings should be interpreted with precaution. Furthermore, the biological mechanism in relation to *APE1* variant, the *APE1* gene expression, and gastric cancer development and metastasis remain to be explored.

In conclusion, our study suggests that the *APE1* T1349G polymorphism may be associated with risk of gastric cancer development in Chinese populations. Large population-based prospective studies with ethnically diverse populations are warranted to verify these findings.
